# End-to-end speech emotion recognition using a novel context-stacking dilated convolution neural network

**DOI:** 10.1186/s13636-021-00208-5

**Published:** 2021-05-12

**Authors:** Duowei Tang, Peter Kuppens, Luc Geurts, Toon van Waterschoot

**Affiliations:** 1 Department of Electrical Engineering (ESAT), STADIUS Center for Dynamical Systems, Signal Processing, and Data Analytics, KU Leuven, Kasteelpark Arenberg 10, Leuven, 3001 Belgium; 2 Faculty of Psychology and Educational Sciences, KU Leuven, Dekenstraat 2, Leuven, 3000 Belgium; 3 e-Media Research Lab, KU Leuven, Andreas Vesaliusstraat 13, Leuven, 3000 Belgium

**Keywords:** End-to-end learning, Speech emotion recognition, Dilated causal convolution, Context stacking

## Abstract

Amongst the various characteristics of a speech signal, the expression of emotion is one of the characteristics that exhibits the slowest temporal dynamics. Hence, a performant speech emotion recognition (SER) system requires a predictive model that is capable of learning sufficiently long temporal dependencies in the analysed speech signal. Therefore, in this work, we propose a novel end-to-end neural network architecture based on the concept of dilated causal convolution with context stacking. Firstly, the proposed model consists only of parallelisable layers and is hence suitable for parallel processing, while avoiding the inherent lack of parallelisability occurring with recurrent neural network (RNN) layers. Secondly, the design of a dedicated dilated causal convolution block allows the model to have a receptive field as large as the input sequence length, while maintaining a reasonably low computational cost. Thirdly, by introducing a context stacking structure, the proposed model is capable of exploiting long-term temporal dependencies hence providing an alternative to the use of RNN layers. We evaluate the proposed model in SER regression and classification tasks and provide a comparison with a state-of-the-art end-to-end SER model. Experimental results indicate that the proposed model requires only 1/3 of the number of model parameters used in the state-of-the-art model, while also significantly improving SER performance. Further experiments are reported to understand the impact of using various types of input representations (i.e. raw audio samples vs log mel-spectrograms) and to illustrate the benefits of an end-to-end approach over the use of hand-crafted audio features. Moreover, we show that the proposed model can efficiently learn intermediate embeddings preserving speech emotion information.

## Introduction

Emotion recognition is a crucial component in present-day human-computer interaction systems. A speech emotion recognition (SER) system utilises vocal expression to recognise emotions and has inherent benefits compared to other modalities. Vocal expression is a fairly direct way to express emotions and is often easier to capture than facial expressions, for which a careful camera positioning is needed. Therefore, an SER system is complimentary to an image/video-based emotion recognition system. Example applications include an SER system intended to analyse the users’ emotions in a call centre to improve their services and an intelligent robot that understands the users’ emotions. Emotion research makes use of both categorical and dimensional approaches to qualify emotional experience. In the categorical approach, discrete emotion labels are used to represent qualitatively different emotional states (e.g. happy, angry). In the dimensional approach, emotional experience is described in terms of a number of basic dimensions, such as valence (ranging from positive to negative) and arousal (ranging from low to high arousal), see [1].

Early SER systems use pre-defined acoustic features to represent the audio recordings. The definition of emotion-related features in this case is a key aspect towards an accurate and robust SER system. Many hand-crafted features have been proposed for this purpose; the Geneva Minimalistic Acoustic Parameter Set (GeMAPS) includes various acoustic features such as frequency-related (e.g. pitch, formant), energy-related (e.g. loudness) and spectral (e.g. spectral slope) features, of which their effectiveness in SER has been evaluated in [2]. Also, the well-known mel-frequency cepstral coefficient (MFCC) features, which have been used in various other speech analysis tasks, including automatic speech recognition, have been applied to SER [3, 4]. However, the design of hand-crafted features requires specialised knowledge, exhaustive selection and massive experiments. Also, the feature extraction process suffers from a potentially huge information loss [5], which could be harmful to the SER performance.

With the rapid developments in deep neural networks (DNNs), the feature extraction for an SER system has shifted to data-driven feature learning. In the area of image processing, convolutional neural networks (CNNs) have been proven to be able to learn abstract features that have intuitively desirable properties while ascending the network layers [6]. Similar work in the area of audio processing has shown that the CNN layers can learn meaningful features by acting as onset extractors, melody extractors, low-pass filters and so on [7]. As a result, the end-to-end learning approach, in which raw microphone recording samples or shallow features are fed into a DNN, becomes feasible and attractive. In the case of SER, this DNN normally consists of both CNN layers and different types of recurrent neural network (RNN) layers [8–15]. The CNN layers are generally applied to the raw recording samples to produce higher-level features. A large receptive field is desired so that the DNN model can receive and learn the long-term temporal information that might be beneficial for SER; as a consequence, the number of parameters in the CNN layers will largely increase when aiming for a larger receptive field.

From the perspective of modelling sequential data, a good model should be able to learn the temporal dependencies or relations within the input sequences. SER in this case has inherent difficulties because the time constants of emotion dynamics can range from just a few seconds to over an hour [16], and these dynamics are regulated by both internal and external excitations [17]. If we assume that the human voice characteristics are a good indication of a person’s internal emotional status, a good SER model should then be able to model sufficiently long temporal dependencies in recorded speech sequences. Many state-of-the-art end-to-end SER systems use long short-term memory (LSTM) layers or gated recurrent unit (GRU) layers as a default network architecture for this purpose [8–15]. However, the RNN type of layers used in the state-of-the-art SER systems suffers from several disadvantages. For example, due to the existence of the recurrent connections, the RNN layer has a sequential type of processing which results in a polynomial growth of computation time with increasing input sequence length. This type of processing is not capable to be parallelised. Also, RNN suffers from the gradient vanishing/exploding problem in processing long sequences, although this problem has been alleviated by the developments in LSTM and GRU [18, 19].

We are aiming to solve these problems that are inherited by the state-of-the-art SER systems from their RNN-type layers, and we will propose a different approach to enlarge the model receptive field without largely increasing its computational complexity. This paper provides details of an improved version of the end-to-end SER model which has been proposed earlier by the authors [20]. The main contributions of this paper can be summarised as follows:

1. We provide more details and propose an updated dedicated dilated convolution block for our neural network model for end-to-end SER. This updated model remains to have a significantly large receptive field while largely reducing the number of model parameters compared to the model proposed in [20].
2. We further explore the context stacking idea, originally proposed in the WaveNet paper [21] and applied to SER in [20], with more thorough comparisons between several model variations.
3. We provide a more in-depth analysis of this new model architecture and evaluate it on both an emotion classification task and an emotion regression task. Abundant simulations have been conducted with two well-known datasets, the REmote COLlaborative and Affective (RECOLA) [22] and the Interactive Emotional Dyadic Motion Capture (IEMOCAP) [23] datasets. Simulation results show that the proposed model surpasses the state-of-the-art CNN-RNN-based models.
4. We also evaluate the effectiveness of end-to-end learning in SER by comparing SER performance using raw audio samples or log mel-spectrogram features with more traditional audio features proposed in earlier work.

The rest of the paper is organised as follows. Section 2 provides a brief overview about the most recent studies related to our work. In Section 3, we introduce the proposed model structure, the parameters of which will be optimised on the concordance correlation coefficient (CCC) objective function [24] for emotion regression. In Section 4, we describe the datasets used and the experimental settings. And then, we will present the simulation results and discuss these in Section 5. Finally, Section 6 presents the conclusions and suggestions for future work.

## Related work

Our proposed SER model has very large receptive field, which makes it suitable for long sequence modelling and is based on the concepts of dilated convolutions, context-stacking, and end-to-end SER.

### Dilated convolution

The dilated convolution is intended to increase the receptive field, which has shown successful outcomes in various audio processing tasks [21, 25]. By stacking many dilated convolution blocks in a DNN, the network will largely increase its receptive field while the model complexity and thus its computational cost will remain reasonably low. Empirical research also shows that this dilated type of convolution outperforms the canonical RNN in various sequence modelling problems [26]. Our work is inspired by the WaveNet model proposed in [21]; however, we propose several modifications to suit the specific application of SER. First, we redesign the dilated causal convolution block inspired by the WaveNet model. Second, we expand the context stacking idea of [21]. Third, we add pooling layers in between the dilated convolution blocks to reduce the sequence length, so that the proposed model is able to deal with very long sequences while posing only moderate memory requirements.

There is hardly any work investigating dilated convolution in an SER framework. In [14], a dilated residual convolution is used to further process the extracted acoustic features. However, the use of the dilated residual convolution in [14] served a different purpose than ours, which in [14], it is to facilitate the reduction of the receptive field and hence yield a strong ability to learn local context. Temporal modelling in [14] was achieved by using LSTM and a self-attention mechanism.

### Context stacking

The context stacking idea was originally proposed in the WaveNet paper [21]. In this idea, multiple trainable DNNs are connected/stacked by local conditioning. More generally, local conditioning has in fact been widely used in various audio-related tasks. In [27], the text-to-speech (TTS) model is conditioned on predicted mel-spectrograms. In another TTS system [28], the model is conditioned on pre-trained speaker embeddings to synthesise speech for a particular person. A similar idea to local conditioning was applied in an SER system [15] by concatenating both the handcrafted acoustic features for SER and the lexical text features to obtain an emotion classifier. Throughout this paper, however, we refer to context stacking only when both the conditioned context and the model itself are trained jointly.

### End-to-end SER

End-to-end learning has attracted vast attention from the deep learning community. The modelling follows a data-driven approach, and little or no specialised knowledge is exploited in this learning process. In audio processing, the traditional learning pipeline (e.g. pre-processing, feature extraction, modelling, inferencing) is taken over by a single DNN, where at the input end raw audio samples or shallow frequency features such as mel-spectrograms are provided, and at the output inference results are obtained (e.g. class labels, parameter estimates).

In SER, Trigeorgis et al. proposed to use a CNN to extract features from the raw audio samples and then use two bidirectional LSTM layers to model the temporal information [8, 9, 11]. Satt et al. afterwards proposed to apply the CNN and the LSTM layers on a modified log-spectrogram, which is harmful for clean SER performance, but it is more robust to noise [10]. In [29], Sarma et al. replaced the CNN layers with time-delay neural network (TDNN) layers. The TDNN tends to increase the receptive field of the network, somewhat resembling the dilated convolution, and was originally used in the speaker recognition problem to extract the x-vector [30].

Some other end-to-end SER systems make use of an attention mechanism which has its origins in natural language processing (NLP) [31]. Chen et al. proposed an SER model consisting of 3-D CNN layers, LSTM layers and the attention layer. This model can learn time-frequency relations from a time stack of log mel-spectrograms [12]. In [15], Li et al. applied a self-attention mechanism along with CNN layers, which can import emotion-salient information from the audio feature inputs. Similarly, in [13, 32], an end-to-end CNN-RNN-based model was combined with the attention mechanism.

### Difference with speaker recognition

To some extent, the emotion classification task resembles the automatic speaker recognition (ASR) problem where the target classes are speaker identities. Similar to SER, the MFCC and frequency-related features (e.g. pitch) are widely used in early ASR research [33, 34]. In [33], Reynolds and Rose propose to use the Gaussian mixture model (GMM) to model the speakers’ MFCC distribution. The concatenation of the mean vectors of the GMM (referred to as the supervector in [33]) can be used to represent the speakers’ identities. This high-dimensional representation is then used to extract the i-vctor, which is a low-dimensional representation of the total variability (i.e. speaker variability and channel variability) [35]. A similar work in SER proposes to extract the emotion representations from the speaker i-vectors [36]. However, in SER, we aim to model very long temporal dependencies of the input features, especially in speech emotion regression, because it is expected that the variation of the features over time is distinguishing for different emotions. This is different from the ASR problem, in which the modelling of the temporal information is less important since the speaker identity is not expected to change within a given speech frame.

## Method

**Fig. 1:**
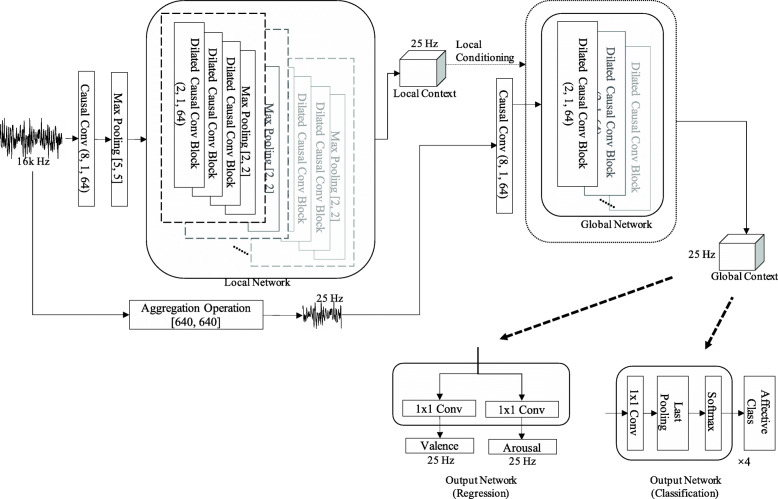
The proposed end-to-end SER model denoted as the dilated-causal-convolution-only speech emotion recognition with context stacking (DiCCOSER-CS). The convolution filter width, stride and filter depth are listed in round brackets, and the pooling width and stride are listed in square brackets

Our proposed end-to-end SER model is shown in Fig. 1, denoted as the dilated-causal-convolution-only speech emotion recognition with context stacking (DiCCOSER-CS). The network consists of dilated causal convolution blocks that are used for increasing the receptive field [21, 37] (Section 3.1), then two sub-networks are stacked and trained jointly (Section 3.2). Finally, the model outputs the arousal and valence estimates for a SER regression task and the class posterior probability for a SER classification task. For regression, the model is trained to minimise the CCC objective function (Section 3.3).

### Dilated causal convolution blocks with local conditioning

**Fig. 2:**
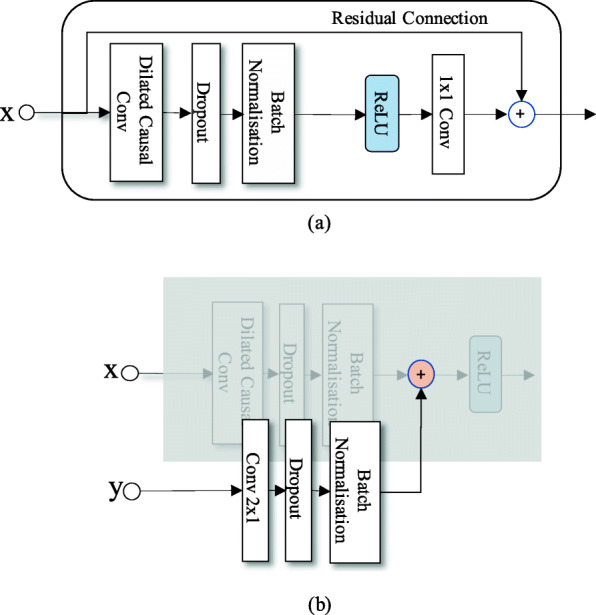
**a** The dilated causal convolution block. **b** The local conditioning

The dilated causal convolution block, shown in Fig. 2a, is one of the basic building blocks in the proposed model. This block is inspired by [21], but from our experiments, we found that using the original dilated causal convolution block in [21] lead to a slow training convergence. Thus, we have redesigned the block in the following aspects. Every dilated causal convolution block consists of two paths, one being the residual connection path and the other being the convolution path. Firstly, the residual path connects the input directly to the output, which has been shown to allow to learn an identity mapping; thus, it can speed up the training and avoid over-fitting [38]. Secondly, in the convolution path, a dilated causal convolution is applied to the input, and it is immediately followed by a dropout layer to prevent the model from over-fitting [39] and a batch-normalisation layer [40] to further speed up the training. Thirdly, we applied the rectified linear unit (ReLU) non-linear activation function [41] to the convolution output. This non-linear activation function has been widely used in modern DNNs for its easy gradient calculation and its effect of giving the model a faster and better convergence. Finally, after going through a 1×1 convolution, the dilated causal convolution path is summed together with the residual path to generate the final output of the block.

We implement the local conditioning similarly to [21]. Consider an input consisting of a sequence of examples, and another sequence ***y*** having the same length as ***x*** containing the conditioning information. If the filter output is ***z***, a local conditioning is defined as follows, adopting the notation from [21]:

1 $$  \boldsymbol{z} = \text{ReLU}(W_{f,k}^{\ast} \boldsymbol{x} + V_{f, k}^{\ast} \boldsymbol{y})  $$

where *W*_*f,k*_,*V*_*f,k*_ are the learnable “filter” parameters in the *k*th layer, ∗ is the convolution operation and ReLU(·) is the ReLU activation function. Dropout and batch normalisation layers are added after the convolution as well, as shown in Fig. 2b.

**Fig. 3:**
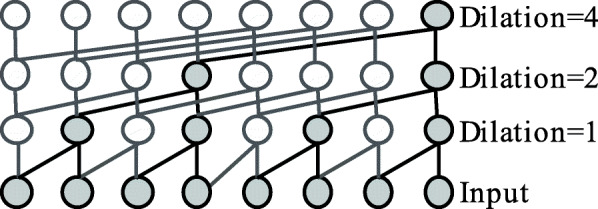
A stack of dilated causal convolution blocks

The dilated causal convolution blocks are then stacked many times with different dilation number in the network. The dilation number defines the time lag, i.e. the number of samples that are skipped in between two input samples used in the convolution with the filter *W*_*f,k*_. Figure 3 shows a stack of dilated causal convolution blocks with filter width 2 and dilation numbers 1, 2 and 4.

### Context stacking using local conditioning

Referring to the context stacking idea in [21], we propose a stacked structure using local conditioning for end-to-end SER, see Fig. 1. This proposed structure consists of three learnable sub-networks. First, one sub-network has a relatively small receptive field, denoted as the “local network”, that receives raw input samples and produces the local context. The local network is capable to be locally conditioned on extra information relating to the input frame (e.g. the speaker gender information, the lexical text information); however, we do not investigate the impact of adding such extra information in this paper. Second, the other sub-network, denoted as “global network”, has a relatively wide receptive field that receives downsampled input audio samples and is aiming to learn global (i.e. long-term) temporal dependencies. The two networks connect by letting the “local network” define the local conditioning on all the layers in the “global network”. We also add pooling layers in the “local network” to downsample the sequence with the aim of reducing computational costs and memory requirements. Finally, the output from the “global network” can be processed by successive convolution-type layers to generate the desired, task-dependent outputs. The reason why we propose to use convolution layers for processing in the final stage is because we are aiming to design a parallelisable network.

### CCC objective function

For a regression task, e.g. an SER model inferencing arousal/valence values, when the training labels are given for very short time intervals (e.g. 40 ms), the levels of affect then can be predicted on the same time scale, i.e. for every 40 ms of a speech recording. In this case, an affective evolution curve can be obtained, e.g. for visualisation purposes. Thus, not only the prediction values should be close to the corresponding ground truth labels, but also the correlation between the entire prediction sequence and label sequence is important. The mean squared error (MSE) or mean absolute error (MAE) loss on the sample level as used in [3] does not consider this correlation. A loss function that has a direct link to the evaluation metric based on the CCC (*ρ*_*c*_) [24] has been proposed in [8, 9, 11].

Given the predicted sequence (denoted by index *m*) of arousal/valence values and its corresponding ground truth sequence (denoted by index *n*), the CCC loss is defined as:

2 $$\begin{array}{*{20}l} \mathcal{L}_{c} &= 1 - \rho_{c} = 1 - \frac{2\rho\sigma_{m}\sigma_{n}}{\sigma_{m}^{2} + \sigma_{n}^{2} + (\mu_{m} - \mu_{n})^{2}} \\&\quad= 1 - \frac{2\sigma_{{mn}}^{2}}{\sigma_{m}^{2} + \sigma_{n}^{2} + (\mu_{m} - \mu_{n})^{2}} \end{array} $$

where *ρ* is the Pearson correlation coefficient (PCC), *μ*_*m*_ and *μ*_*n*_ are the sample means, $\sigma ^{2}_{m}$ and $\sigma ^{2}_{n}$ are the sample variances and $\sigma ^{2}_{{mn}}$ denotes the covariance between the two sequences. Therefore, prediction sequences exhibiting a weak correlation with the ground truth sequences as well as shifted amplitude of the prediction values are both penalised in one loss function.

## Experimental set-up

### Implementation details

In this section, we describe how to implement the DiCCOSER model. The raw audio samples are firstly fed into a causal convolution layer which has a filter width equal to 8. The filter width of this convolution layer on one hand should be wider than the successive pooling size so that it has a sufficiently large receptive field and is capable of extracting salient features which will be selected by the following pooling operation, and on the other hand, the filter width should be kept small to maintain a low model complexity. This causal convolution layer maps the single-channel audio sample sequences to 64-dimensional vector sequences. Next, these sequences are downsampled in a max-pooling layer with size 5, and are then ready for the processing by the “local network”. The configuration of the pooling size is task-dependent, such that after the pooling operations, the output sequence has the desired sampling rate.

Stride one and zero-padding is used across all convolution layers in the entire network to retain the same sequence length after each operation.

#### Local network

Firstly, we construct the local network, which contains a stack of dilated causal convolution blocks. These blocks have a filter depth equal to 64, and their dilation numbers are chosen to correspond to a subset of a geometric series and are repeated a few times. Max-pooling layers are included after every stack of dilated causal convolution blocks, and the number of max-pooling layers is task-dependent. Specifically, we design each stack in our local network to have a set of dilation numbers *D*^local^ ={2^*k*^,*k*=0,1,2}, and a total of 7 such stacks are used. There is one pooling-size-2 max-pooling layer after each stack of dilated causal convolution blocks, which results in 7 pooling layers in total. The pooling layers in the local network progressively downsample the processed data from 16 kHz at the input to 25 Hz at the output of the local network, which is the same as the label sampling rate for the regression task.

In parallel to the local network, the raw audio input sequence is directly downsampled from 16 kHz to 25 Hz. We propose two aggregation operations that extract useful features from the signal frames in this parallel feedforward branch: (a) a *max-pooling aggregation* that extracts the maximum value from the frames and (b) an *RMS aggregation* that calculates the RMS value per frame. We believe that these features can be representative of the original audio frames at the reduced sampling rate of 25 Hz, and they are also related to the expression of emotions [42]. The downsampled sequences are finally mapped to 64-dimensional vector sequences by a causal convolution layer with filter size 8.

#### Global network

Secondly, in the global network, the dilated causal convolution blocks have filter depth equal to 64 as well. The aim of the global network is to learn the long-term temporal dependencies from a more global perspective. It can ensure this aim by processing the downsampled input sequences because this not only reduces the computational cost of the global network, but also prevents the global network from attempting to model subtle changes in the original raw input samples. In addition, thanks to the context-stacking structure, some information lost in the downsampling operation is selectively passed to the global network. In order to be able to effectively perform the dilated convolutions, we propose to have the largest dilation number equal to the length of the processed input frames. We set the global network dilation numbers as $D_{\text {global}} =\{1, 2, 2^{2}, \dots, 2^{\lfloor \text {log}_{2}(L)\rfloor }, L\}$, where *L* is the input frame length and ⌊·⌋ denotes the flooring operation. For example, for a 20-s audio input frame, the global network operates on the downsampled 25-Hz sequence of length 500, such that *D*_global_={1,2,4,8,16,32,64,128,256,500}.

Finally, all the dilated causal convolution blocks are conditioned on the local contexts. We implement these context stacking filters (filter “ *V*_*f,k*_” in (1)) by normal CNN layers with filter width 2 and filter depth 64.

#### Output network

Finally, the output network converts the global network output to the desired output formats. In our regression task, there are two 1×1 convolution layers with filter depth 1 in the output network that yield the arousal and valence predictions.

In our classification task, there are four 1×1 convolution layers with filter depth 1 corresponding to 4 different class outputs. These are then processed by a last-pooling layer to only keep the last convolution output for every layer. This is because our model has a receptive field as large as the input sequence length, so that the last convolution output can be trained to contain global information. Finally, a softmax layer is applied, and the output of the softmax layer can be interpreted as the class posterior probability.

### Datasets

To evaluate the regression and classification performance of the proposed model and to compare it with the state-of-the-art end-to-end CNN-LSTM-based SER model, we used two widely used affectively labelled datasets: the RECOLA dataset [22] for the regression task and the IEMOCAP dataset [23] for the classification task.

#### RECOLA

The RECOLA dataset [22] contains abundant affective data with both arousal and valence annotations per 0.04 s. The arousal and valence annotations are continuous values in the range [ −1, 1]. The speech data consist of interviews in which people talk about real-life stories. However, since the database is not fully publicly available, we can only acquire a sub-partition that is used in the 2015 and 2016 Audio/Visual Emotion Challenge and Workshop (AVEC) [43, 44] competitions. We only use the raw audio out of four modalities (audio, video, electrocardiogram (ECG) and electro-dermal activity (EDA)) provided by the competition and their corresponding labels. This sub-partition contains eighteen 5-min-long audio clips, equally divided into a training set and a development set. In both sets, there are 5 clips with female speakers and 4 clips with male speakers, as indicated in Table 1. The language of all audio clips is French. The sampling frequency is 44.1 kHz, and we downsampled all data to 16 kHz for our simulations. We further divide this sub-partition into 5-folds. The partitions are summarised in Table 2.

**Table 1:** RECOLA database sub-partition naming and speaker gender in brackets

Train	train_1 (M)	train_2 (F)	train_3 (M)	train_4 (F)	train_5 (F)
	train_6 (M)	train_7 (F)	train_8 (F)	train_9 (M)	
Dev	dev_1 (F)	dev_2 (M)	dev_3 (M)	dev_4 (M)	dev_5 (F)
	dev_6 (M)	dev_7 (F)	dev_8 (F)	dev_9 (F)

**Table 2:** RECOLA database 5-fold cross-validation partitions

	Fold 1	Fold 2	Fold 3	Fold 4	Fold 5
Valid	train_1, dev_1	train_3, dev_3	train_5, dev_5	train_7, dev_7	train_9
Test	train_2 dev_2	train_4, dev_4	train_6, dev_6	train_8, dev_8	dev_9

#### IEMOCAP

The IEMOCAP dataset [23] contains English acted speech dialogues by 10 professional actors. There are in total 5 sessions, each featuring one actor and one actress performing the dialogues with a script or in an improvised manner. There is no speaker overlap across the 5 sessions. We have used improvised utterances from four emotional categories {neutral, anger, sadness and happiness}, which in total are 2280 utterances (neutral 1099, anger 289, sadness 608, happiness 284). We trim or pad zeros in front of the recordings to make them into 3-s-long clips. We divide the cross-validation folds by the session numbers (i.e. session 1 to 5). The audio files have also been downsampled to 16 kHz.

#### Data augmentation

To overcome over-fitting, we propose to apply two simple yet distinct data augmentation methods to the regression task and the classification task respectively. For the regression task, we propose to use a sliding window data augmentation, that is, we use a 20-s-long sliding window to generate the training frames from the original 5-min recordings. Successive sliding windows are shifted by 4 s.

For the classification task, instead of using speed perturbation as in [45], which we believe may change the affective expression of the recording, we propose to use a random flipping data augmentation. That is, with a given probability (e.g. equal to 50%), the input sequence is flipped (i.e. time reversed) before being fed into the model. This data augmentation method will largely retain the affective meaning of the original speech recording and will prevent the model to become biassed by some disturbing context factors having a specific temporal pattern or dependence (e.g. room reverberation).

#### Training and evaluation settings

For the regression task, we optimise the model by minimising the CCC loss as described in Section 3.3, and for the classification task, we minimise the cross-entropy loss. The RMSProp optimiser [46] is used to train the models, with a fixed learning rate of 10^−4^. The batch size is 5 for the regression task and 10 for the classification task, due to memory constraints. *l*_2_ regularisation with a regularisation parameter of 10^−4^ is applied, and the dropout rate equals 0.5. No post-processing is performed on the output predictions.

We conduct all the experiments using 5-fold cross-validation. More specifically, for the RECOLA dataset, in every run, we use 4-folds of data to train the model, then we split the last fold into a validation set and a testing set as listed in Table 2. In this way, there is no speaker overlapping across the three subsets. For the IEMOCAP dataset, since we aim to evaluate the model’s generalisability across speakers and sessions, for each run, we use 3-folds for training, and the remaining 2-folds for validation and testing respectively. In each experiment, we employ early stopping, i.e. we stop the training on the highest validation performance, and then use that model to compute the testing performance.

The CCC [24] is used to evaluate the regression task, whereas the weighted accuracy (WA) and the unweighted accuracy (UA) are used to evaluate the classification task. WA is the overall accuracy of the entire testing data, which indicates the overall model performance across all classes, whereas UA is the average accuracy for each emotion class, which marginalises out the effect of the existence of class imbalance. All the experiments are repeated 5 times using data from different folds as stated above. The results are averaged across the 5-folds before being reported.

## Results and discussions

### Evaluation of the context-stacking idea and the model architectures

First, we evaluate the context stacking which is considered as one of the contributions of this paper to SER, and a few model architecture variations. More specifically, we aim to answer the following questions:

1. Can the context-stacking architecture increase the model performance?
2. What is the optimal choice between the max-pooling aggregation and the RMS aggregation?

**Fig. 4:**
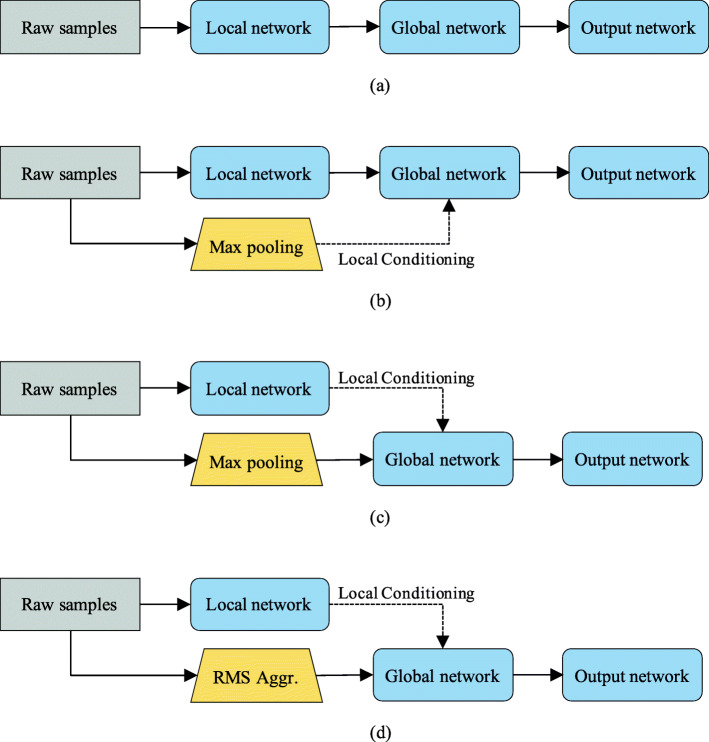
The proposed model variations. **a** DiCCOSER. **b** DiCCOSER-CS-V2. **c** DiCCOSER-CS max. **d** DiCCOSER-CS rms. “RMS Aggr.” indicates using the RMS aggregation
- A model that deploys context stacking, but the local contexts are directly fed to the global network, and the global network is locally conditioned on the downsampled input sequences (denoted as DiCCOSER-CS-V2 max), is shown in Fig. 4b. In addition, the max-pooling aggregation is used.
- The proposed model that deploys context stacking as described in Sections 4.1.1 and 4.1.2 (i.e. the global network receives the aggregated downsampled input sequences and context stacking is performed using the local contexts), and uses the max-pooling aggregation method in the downsampled stream (denoted as DiCCOSER-CS max), is shown in Fig. 4c.
- The proposed model having similar architecture as the model variation c), but using the RMS aggregation method (denoted as DiCCOSER-CS rms), is shown in Fig. 4d.

We propose and evaluate four model variations, and their topologies are shown in Fig. 4:

- A model that contains only one sequential network (denoted as DiCCOSER), in which the context stacking is not used, is shown in Fig. 4a.

All the model variations have dilation numbers in the local network and the global network as described in Sections 4.1.1 and 4.1.2.

**Fig. 5:**
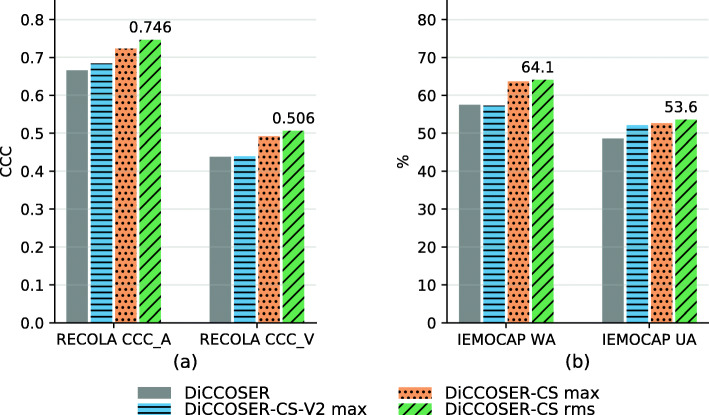
Performances of four proposed model variations on **a** the RECOLA dataset and **b** the IEMOCAP dataset. Suffix “-CS” indicates using the context stacking; “max” and “rms” indicate using the max-pooling aggregation and the RMS aggregation, respectively

We evaluate the proposed model variations on both the regression task (on the RECOLA dataset) and the classification task (on the IEMOCAP dataset). The average testing results for both tasks are illustrated in Fig. 5. First, we can see that the model variations with context stacking (i.e. the DiCCOSER-CS-V2 max, the DiCCOSER-CS max and the DiCCOSER-CS rms variations) perform better than the DiCCOSER model that does not use context stacking in both tasks. Compared to the DiCCOSER model, the DiCCOSER-CS max variation improves arousal CCC, valence CCC, WA and UA with about 8.5%, 12.3%, 10.7% and 8.2%, respectively, and the DiCCOSER-CS rms variation improves arousal CCC, valence CCC, WA and UA with about 12%, 15.5%, 11.5%, and 10.3%, respectively. On the other hand, the improvements for the DiCCOSER-CS-V2 model on the RECOLA dataset are not significant (it improves arousal CCC and valence CCC with about 2.7% and 0.5%, respectively), and the DiCCOSER-CS-V2 model improves UA on the IEMOCAP dataset with about 7.2%, but shows a small degradation in WA (about 0.3%). Second, in the case with context stacking, the model with the RMS aggregation method performs better than the model with the max-pooling method, and both DiCCOSER-CS variations perform better than the DiCCOSER-CS-V2 variation. However, the UA difference between the variations DiCCOSER-CS-V2 max (UA equal to 52.1%) and DiCCOSER-CS max (UA equal to 52.7%) is not large. Overall, the best performance is obtained with the DiCCOSER-CS rms model. In the regression task, its arousal CCC equals 0.746, and its valence CCC equals 0.506. In the classification task, the DiCCOSER-CS rms model achieves a WA equal to 64.1% and an UA equal to 53.6%.

We can conclude that the context-stacking architecture does improve the SER performance. Also, the classification performance of the DiCCOSER-CS max and the DiCCOSER-CS rms variations shows a similar trend in WA and UA, which indicates that the improved accuracy is not due to the bias towards an individual class. Furthermore, the RMS aggregation method works better than the max-pooling method, so we keep using the DiCCOSER-CS rms model for the further experiments.

### Comparisons with the state-of-the-art model in the speaker-independent setting

**Fig. 6:**
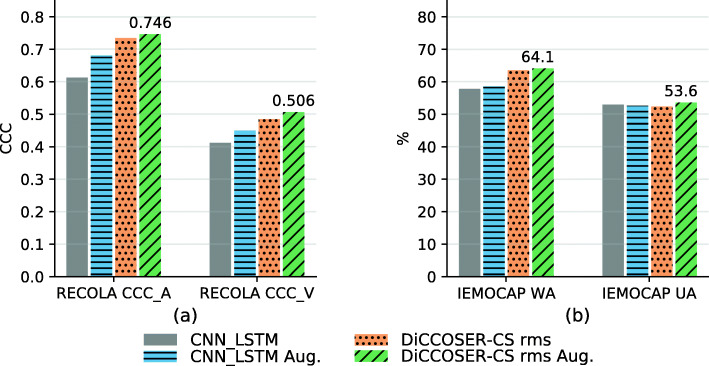
Performance comparisons to the baseline CNN-LSTM model. “Aug.” indicates using the sliding window augmentation in **a** or the random flipping augmentation in **b**

In the second experiment, we compare our proposed model with the state-of-the-art CNN-LSTM model. In our dataset partitions, in each fold, the speakers in the training, validation and testing sets are not overlapping, so that the model performance is speaker-independent. The baseline model proposed by Tzirakis et al. [11] was originally proposed for the emotion regression task on the RECOLA dataset and consists of three CNN layers with max-pooling and dropout layers in between and subsequently two LSTM layers. The model has been implemented here with settings as stated in [11], and in the classification task, we have added a global average pooling layer and a softmax layer on the outputs of the last LSTM layer of the baseline model. This model is optimised on the CCC loss for the regression task, and on the cross-entropy loss for the classification task, identically to our proposed model. Lastly, we also evaluate the baseline model trained with the proposed data augmentation methods (the sliding window augmentation and the random flipping augmentation method, denoted as “Aug.”), which was not proposed in their original paper [11]. To have fair comparisons, we also conduct experiments using our proposed model without data augmentation. The results are shown in Fig. 6.

The results firstly illustrate that the proposed model, with the proposed data augmentation methods, outperforms the CNN-LSTM model with or without data augmentation in all the testing cases. More specifically, in the regression task, the DiCCOSER-CS rms model with sliding window augmentation achieves arousal CCC equal to 0.746 and valence CCC equal to 0.506, compared to the CNN-LSTM model with sliding window where arousal CCC is 0.681 and valence CCC is 0.449. Hence, the DiCCOSER-CS rms model with sliding window has improved the arousal CCC and valence CCC with 9.5% and 12.7%, respectively. In the classification task, the DiCCOSER-CS rms model with random flipping augmentation achieves a WA equal to 64.1% and a UA equal to 53.6%, whereas the CNN-LSTM model with random flipping augmentation yields WA and UA equal to 58.6% and 52.6%, respectively. Secondly, the proposed data augmentation methods can improve the SER performance for both the baseline CNN-LSTM model and the proposed DiCCOSER-CS rms model. More specifically, with regard to the regression task, the sliding window augmentation improves the CNN-LSTM model performance from 0.613 to 0.681 on arousal CCC and from 0.412 to 0.45 on valence CCC. Also, it improves the DiCCOSER-CS rms model performance on arousal CCC from 0.734 to 0.746 and valence CCC from 0.484 to 0.506. However, in the classification task, the random flipping augmentation only gently improves the WA performance (the CNN-LSTM model with augmentation improves WA from 57.8 to 58.6%, and the DiCCOSER-CS rms model with augmentation improves WA from 63.5 to 64.1%), and also the UA of the DiCCOSER-CS model (from 52.3 to 53.6%), but there is a small decrement in the WA of the CNN-LSTM (about 0.5%). Nevertheless, these results may indicate that these data augmentation methods are helpful to improve the SER testing performance.

Finally, an interesting comparison on the number of parameters is given in Table 3. It is shown that even if the proposed DiCCOSER-CS model has the best overall performance on both the regression and classification tasks, it only has about 1/3 of the number of parameters compared to the baseline CNN-LSTM model. The low number of parameters implies that the proposed model can be processed much faster in both training and inferencing even with a single thread.

**Table 3:** Summary of the number of model parameters

	CNN-LSTM [11]	DiCCOSER-CS RECOLA	DiCCOSER-CS IEMOCAP
Number of parameters	≈1300·10^3^	≈475·10^3^	≈430·10^3^

### Comparisons with different input features

In this experiment, we aim to evaluate the impact of using different input features on the SER performance. More specifically, we conduct experiments using log mel-spectrogram features in both the baseline CNN-LSTM model and the proposed DiCCOSER model, since log mel-spectrogram features are widely used in end-to-end SER[10, 12, 32]. The results are then compared with the aforementioned models which use raw time domain audio samples as an input. Because the log mel-spectrogram features have a much lower sampling rate than the raw audio samples, we modify the baseline CNN-LSTM model and our proposed DiCCOSER model with/without context stacking to have less pooling layers and a smaller pooling width to work with these features. It is worth to mention that, in the case of using the DiCCOSER-CS model, we only change the local network to receive the log mel-spectrogram features, in which the local context is generated to be used by the global network via local conditioning. The global network still uses the aggregated raw samples as an input. To extract the log mel-spectrogram features, we use a short-time Fourier transform (STFT) with a window size equal to 0.04 s, 50% overlap and 40 mel-frequency bands in the range of [0, 7600] Hz. Finally, we use the logarithmic values for numerical stability.

**Fig. 7:**
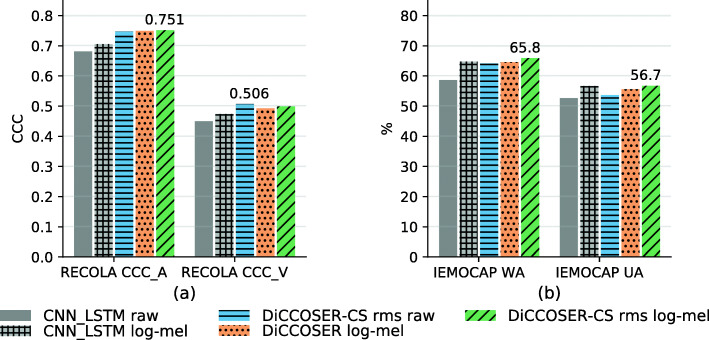
Comparisons between using raw time domain samples and using log mel-spectrograms as the SER model input

The results are illustrated in Fig. 7. We can see that using the log mel-spectrogram features can improve the SER performance of the CNN-LSTM model; however, the improvements for the DiCCOSER models are less pronounced. Firstly, with regard to the regression task on the RECOLA dataset in Fig. 7a, using log mel-spectrogram features significantly improves the CNN-LSTM model performance (arousal CCC and valence CCC increase with about 3.5% and 5.1%, respectively). Similarly, the CNN-LSTM model with log mel-spectrogram features also largely improves the WA and UA on the IEMOCAP dataset (WA and UA increase with 10.4% and 7.6%, respectively). This makes the performance of the CNN-LSTM model with log mel-spectrogram features comparable to the performance of some of the proposed DiCCOSER models. Its UA achieves 56.6% which is higher than the DiCCOSER-CS rms model with raw sample inputs (UA equal to 53.6%) and is higher than the DiCCOSER model with log mel-spectrogram features (UA equal to 55.5%). The best WA (equals to 65.8%) and UA (equal to 56.7%) are however still obtained with the DiCCOSER-CS rms model with log mel-spectrogram input.

Secondly, for the DiCCOSER models, there is no obvious difference among using the raw audio samples or the log mel-spectrogram features as an input. Regarding the performance for the regression task on the RECOLA dataset, the best arousal CCC (equal to 0.751) is obtained with the DiCCOSER-CS rms model with the log mel-spectrogram input, which is slightly higher than the DiCCOSER-CS rms model with the raw sample input (arousal CCC equal to 0.746), and is slightly higher than the DiCCOSER model with log mel-spectrogram input (arousal CCC equal to 0.749). Analogously, with respect to valence CCC, the three model variations perform equally well (valence CCC from high to low: DiCCOSER-CS rms model with raw audio sample input gives a valence CCC equal to 0.506, the DiCCOSER-CS rms model with log mel-spectrogram input gives a valence CCC equal to 0.498 and the DiCCOSER model with log mel-spectrogram input gives a valence CCC equal to 0.492). Similar conclusions can be drawn from the classification results on the IEMOCAP dataset, although the log mel-spectrogram features slightly improve the DiCCOSER model and the DiCCOSER-CS model performance, especially in UA, which may indicate that using log mel-spectrogram input features provides higher robustness to class imbalance than the raw audio input, but there is no apparent winner among the DiCCOSER variations. The best results for the classification task are obtained with the DiCCOSER-CS rms model using the log mel-spectrogram features (WA equal to 65.8% and UA equal to 56.7%).

Thirdly, the conclusions from the previous experiments are still valid, i.e. the DiCCOSER model with context stacking performs slightly better than the DiCCOSER model without context-stacking, and the DiCCOSER-CS rms model outperforms the CNN-LSTM baseline model when the same input features are used.

Finally, we also compare our end-to-end SER performance with some existing SER models that use traditional audio features. The comparisons are listed in Table 4. Sahu et al. [47] and Jiang et al. [48] have evaluated the GeMAPS features on the IEMOCAP dataset, and Jiang et al. [48] have evaluated MFCCs as well. The results indicate that the end-to-end learning models (including the baseline CNN-LSTM model [11] and the proposed DiCCOSER models), using time-domain raw audio samples or shallow features such as the log mel-spectrogram features, outperfom the models using traditional audio features. Similarly with the RECOLA dataset, the CNN-LSTM model and the proposed DiCCOSER model with raw or shallow features (i.e. log mel-spectrogram features) outperform the traditional audio features (such as the GeMAPS or low-level descriptor features evaluated in Valstar et al. [44] and Ringeval et al. [3], respectively) in terms of the mean CCC performance.

**Table 4:** Performance comparisons to SER models trained using traditional audio features; best performances are in bold

	RECOLA			IEMOCAP	
	CCC_A	CCC_V	CCC Mean	WA	UA
Sahu et al. [47] (GeMAPS)	-	-	-	56.85%	-
Jiang et al. [48] (GeMAPS)	-	-	-	-	41%
Jiang et al. [48] (MFCCs)	-	-	-	-	35%
Valstar et al. [44] (GeMAPS)	0.683	0.375	0.529	-	-
Ringeval et al.[3]					
(low-level descriptors)	**0.757**	0.26	0.509	-	-
CNN-LSTM Tairakis et al. [11]					
(raw samples)	0.681	0.5	0.591	58.6%	52.6%
CNN-LSTM Tairakis et al. [11]					
(log mel-spectrogram)	0.705	0.473	0.589	64.7%	56.6%
Proposed DiCCOSER-CS					
(raw samples)	0.746	**0.506**	**0.626**	64.1%	53.6%
Proposed DiCCOSER-CS					
(log mel-spectrogram)	0.751	0.498	0.6245	**65.8**%	**56.7%**

### Visualisations of the local contexts

**Fig. 8:**
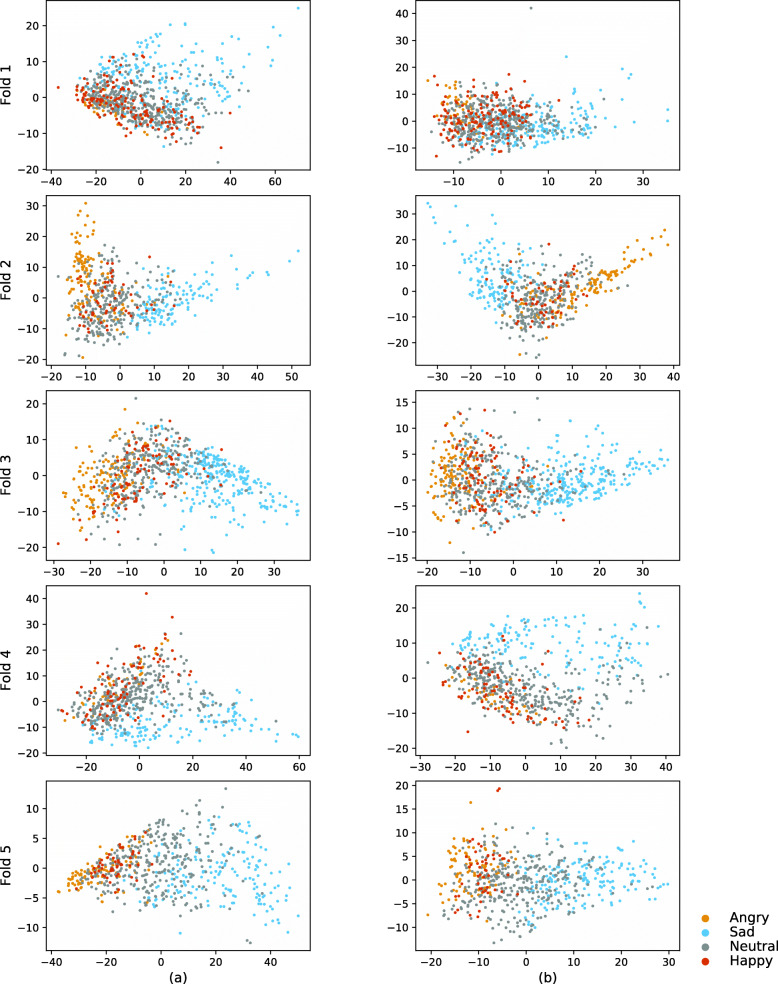
Visualisations of the local contexts of the testing sets from the IEMOCAP folds

In this experiment, we aim to investigate the local contexts produced by the proposed SER model. Ideally, the local contexts should learn the information that has been lost during the aggregation step applied to the input sequence in the parallel feedforward branch. To this end, we visualise the local contexts computed from the IEMOCAP test sets in different cross-validation folds. These local contexts are originally 64-dimensional, but for visualisation purposes, here, we first average them across time per input audio frame, then we compute their first 2 principal components using principal component analysis (PCA). In Fig. 8, the horizontal axis corresponds to the first principal component, and the vertical axis corresponds to the second principal component. Finally, we assign colours to the local contexts corresponding to their class labels. Columns (a) and (b) in Fig. 8 are corresponding to the DiCCOSER-CS model with the max-pooling aggregation and with the RMS aggregation, respectively.

We can make the following observations from the visualisations of the local contexts. First, we can see that the local contexts tend to form clusters that correspond to the class labels. The most discriminative cluster is the cluster corresponding to “Sad” (in light blue), and another discriminative cluster is the “Angry” cluster (in yellow). The “Neutral” and “Happy” clusters are overlapping in most of the cases. This can be explained by the fact that the angry emotion has a very high energy (high arousal), and the sad emotion has a very low energy (low arousal) which are both more distinguishable than the happy (medium to high energy/arousal) and neutral emotion. Second, we found that the first principal component represents the arousal properties of the emotions. That is, since arousal indicates the activation/energy of an emotion [1], if we look at the emotion clusters along the first principal component axis (from left to right along the horizontal axis), these emotion clusters are arranged from low activated emotion clusters to high activated emotion clusters or the other way around. Thus, the first principal component of the local context is actually highly positively or negatively correlated with the arousal, even if we only supervise the training with class labels (i.e. categorical labels such as sad, happy, angry and neutral). However, the second principal component of the local contexts does not show a significant correlation to the valence, which indicates the pleasurableness of an emotion. Finally, we observe that the local contexts learned from the DiCCOSER-CS model with the max pooling aggregation and with the RMS aggregation show similar geometrical properties in this low-dimensional PCA space. In other words, the points from the same test set are spread similarly even when using different aggregation methods, and in particular, they form similar shapes in this low-dimensional space but mirrored along the first or second principal component axis.

## Conclusions and future work

In this work, we have proposed a novel end-to-end DNN model for SER that does not consist of any recurrent or fully connected layers. A dedicated dilated causal convolution block is designed to increase the model receptive field while keeping the number of model parameters low. Simulation results firstly indicate that the proposed model with context stacking and the RMS aggregation method achieves the best SER performance among several model variations, which confirms the effectiveness of the novel context stacking structure for SER. Secondly, the simulation results also indicate that the proposed model variations, which only contained about 1/3 of the number of model parameters, outperformed the baseline state-of-the-art CNN-LSTM model. Thirdly, we have shown that the proposed sliding window augmentation and random flipping augmentation methods improve the SER performance for both the baseline model and the proposed model. Fourthly, using log mel-spectrogram features instead of raw audio samples as an input can significantly improve the CNN-LSTM model SER performance and slightly improves the proposed model SER performance, which indicates that the log mel-spectrogram features can be alternative input features for end-to-end SER. Furthermore, by using either the raw audio samples or the shallow log mel-spectrogram features as an input, the baseline model and the proposed model both achieve better SER performance compared to the SER systems using traditional audio features. Finally, we reported the results that allow to interpret the local contexts. We found that (1) the local contexts form clusters that corresponded to their emotion class labels, which indicated that the local contexts tend to learn the affective information not included in the downsampled input sequence; (2) the first principal component of the local context is highly positively or negatively correlated with the arousal of the target emotion, even if we only supervised the model training with the emotion class label.

Future work could investigate using several parallel local networks operating on different input hop lengths, similarly to the idea in [49], which is aiming to learn the local contexts from the input at different levels of compression. In addition, it appears highly attractive to apply the dilated causal convolution to similar tasks where a large receptive field is required, for example, by using the proposed architecture in an autoencoder structure to learn emotion representations from raw speech signals or applying the proposed architecture to other speech-related problems.

## Data Availability

The datasets generated and/or analysed during the current study are available in the IEMOCAP repository (https://sail.usc.edu/iemocap) and the RECOLA repository (https://diuf.unifr.ch/main/diva/recola). However, restrictions apply to the availability of these data, which were used under licence for the current study, and so are not publicly available. Data are however available from the authors upon reasonable request and with permissions of the SAIL Lab at the University of Southern California and the RECOLA group at the Université de Fribourg. Declarations

## Abbreviations

SER: Speech emotion recognition
DNN: Deep neural network
CNN: Convolutional neural network
GPU: Graphical processing unit
RECOLA: REmote COLlaborative and Affective
AVEC: Audio/Visual Emotion Challenge and Workshop
RNN: Recurrent neural network
LSTM: Long short-term memory
GRU: Gated recurrent unit
CCC: Concordance correlation coefficient
TTS: Text-to-speech
GeMAPS: Geneva Minimalistic Acoustic Parameter Set
MFCC: Mel-frequency cepstral coefficient
IEMOCAP: Interactive emotional syadic motion capture
TDNN: Time-delay neural network
NLP: Natural language processing
MSE: Mean squared error
MAE: Mean absolute error
PCC: Pearson correlation coefficient
ReLU: Rectified linear units
DiCCOSER-CS: Dilated-causal-convolution-only speech emotion recognition with context stacking
WA: Weighted accuracy
UA: Unweighted accuracy
PCA: Principal component analysis
STFT: Short-time Fourier transform
